# Hybrid Weld-Bonded Joints: A Critical Comparative Review of Welding Processes, Adhesive Interaction and Joint Performance

**DOI:** 10.3390/ma19112288

**Published:** 2026-05-28

**Authors:** Anna Krawczuk

**Affiliations:** Department of Machine Operation and Production Processes Management, University of Life Sciences in Lublin, Głęboka 28, 20-612 Lublin, Poland; anna.krawczuk@up.edu.pl

**Keywords:** weld-bonded joints, hybrid joining, resistance spot weld-bonding, friction stir weld-bonding, structural adhesives, mechanical performance

## Abstract

Weld-bonded joints combine localized metallic welding with structural adhesives and are increasingly used in lightweight multi-material structures. Although numerous studies have examined individual weld-bonding processes, the available literature remains fragmented with respect to process classification, adhesive–weld interaction and mechanical performance. This paper presents a critical review of hybrid weld-bonded joints published between 2000 and 2026, with emphasis on welding-based joining processes and their influence on joint behavior. The main weld-bonding techniques, including resistance spot weld-bonding (RSWB), friction stir weld-bonding (FSWB), friction stir spot weld-bonding (FSSWB) and laser weld-bonding (LWB), are systematically compared in terms of heat input, adhesive stability, load transfer mechanisms and mechanical performance. The analysis indicates that processes with lower heat input, such as FSWB and FSSWB, provide improved adhesive preservation and fatigue performance, whereas RSWB remains the most industrially established solution. The influence of different adhesive families (epoxy, polyurethane, acrylic and thermoplastic) is evaluated with respect to thermal resistance, rheological behavior during welding and long-term durability. Mechanical performance under static, fatigue and impact loading is critically assessed, highlighting typical strength improvements compared with purely welded joints and identifying dominant failure modes. In addition, numerical modeling approaches, including finite element and cohesive zone methods, are reviewed in terms of their ability to capture coupled thermomechanical and damage phenomena. The review further outlines key industrial applications, current technological limitations and future research directions, including advanced adhesive systems, low-heat-input processes, non-destructive testing and digital-twin-based optimization.

## 1. Introduction

Joining technologies play a key role in modern industries, particularly in the production of lightweight multi-material structures in automotive and aerospace applications [1,2,3,4]. Welding, adhesive bonding and mechanical joining are commonly used to combine high-strength steels, aluminum alloys and composite materials, enabling weight reduction while maintaining structural performance and safety [5,6,7,8]. Among these, resistance spot welding (RSW) remains the dominant technique in automotive body manufacturing due to its high productivity and ease of automation [9,10], whereas friction-based processes, such as friction stir welding (FSW), are increasingly applied in aerospace structures to reduce thermal distortion and microstructural degradation [11,12].

Adhesive bonding is another widely used joining method, enabling uniform load transfer and effective joining of dissimilar materials, including metals, polymers and composites [13,14,15]. Compared with conventional welding, adhesive joints provide improved stress distribution, vibration damping and corrosion protection [16,17]. However, their performance is limited by relatively low resistance to elevated temperatures, reduced peel strength and sensitivity to surface preparation and environmental conditions [18,19].

**Figure 1:**
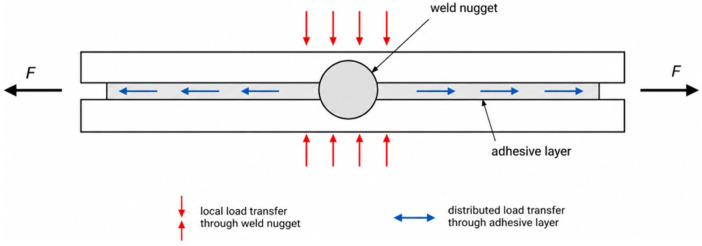
Conceptual schematic of a hybrid weld-bonded joint showing local load transfer through the weld nugget and distributed load transfer through the adhesive layer.

To overcome the limitations of individual techniques, hybrid joining methods have been developed, combining two or more joining approaches within a single joint [4,6,20]. Among these, weld-bonded joints represent a particularly important solution, integrating metallic welding with structural adhesives [21,22]. This hybrid configuration enables a synergistic load transfer mechanism, in which the weld provides localized load-bearing capacity, while the adhesive layer ensures more uniform stress distribution. As illustrated in Figure 1, load is transferred locally through the weld nugget and more gradually through the adhesive layer. As a result, weld-bonded joints are increasingly applied in automotive, aerospace and rail industries, where they contribute to weight reduction, improved structural stiffness and enhanced durability [14,16,22].

Weld-bonding techniques include several process variants, such as resistance spot weld-bonding (RSWB), friction stir weld-bonding (FSWB), friction stir spot weld-bonding (FSSWB) and laser weld-bonding (LWB) [23,24,25,26]. These processes differ significantly in terms of heat input, adhesive interaction and resulting joint properties. In particular, the thermal conditions during welding strongly influence adhesive degradation, load-transfer mechanisms and long-term performance. Therefore, understanding the interaction between welding processes and adhesive behavior is essential for optimizing hybrid joint design.

Although weld-bonding has been studied for several decades, existing research remains fragmented. Most published studies focus on individual processes or selected aspects, such as microstructure, fatigue performance or numerical modeling [8,21,27]. As a result, there is still a lack of a unified and comparative framework enabling direct comparison between different weld-bonding techniques.

Unlike previous studies limited to single process types, this paper adopts a unified and comparative approach, enabling direct analysis of resistance, friction-based and laser weld-bonding techniques within a common framework. This paper presents a comprehensive and critical review of hybrid weld-bonding technologies, including process classification, adhesive–weld interaction, mechanical performance and industrial applications. Particular emphasis is placed on the influence of welding processes on joint behavior and on identifying key relationships between process conditions, adhesive properties and structural performance. To the best of the authors’ knowledge, such a unified comparative framework has not been systematically presented in the existing literature.

The review covers scientific publications from 2000 to 2026 related to weld-bonding technologies based on resistance, friction, and laser welding. The analyzed studies include both experimental and numerical investigations of mechanical performance, microstructural evolution, thermal phenomena, and the durability of hybrid joints. This approach enables a consistent comparison of different weld-bonding processes and provides a basis for identifying current limitations and future research directions in the field of hybrid joining technologies. Furthermore, this review proposes a comparative framework that links welding heat input, adhesive behavior, and the mechanical performance of weld-bonded joints.

## 2. Definition and Classification of Hybrid Joints

Hybrid joining technologies are increasingly applied in lightweight engineering structures because they combine different load transfer mechanisms within a single joint configuration. By integrating welding, adhesive bonding or mechanical fastening, hybrid joints may provide enhanced load distribution, higher damage tolerance and improved structural performance compared with single-method joining techniques [4,5,6,8]. Such solutions are particularly attractive for lightweight multi-material structures used in automotive, aerospace and transportation and lightweight engineering applications [16,20].

**Figure 2:**
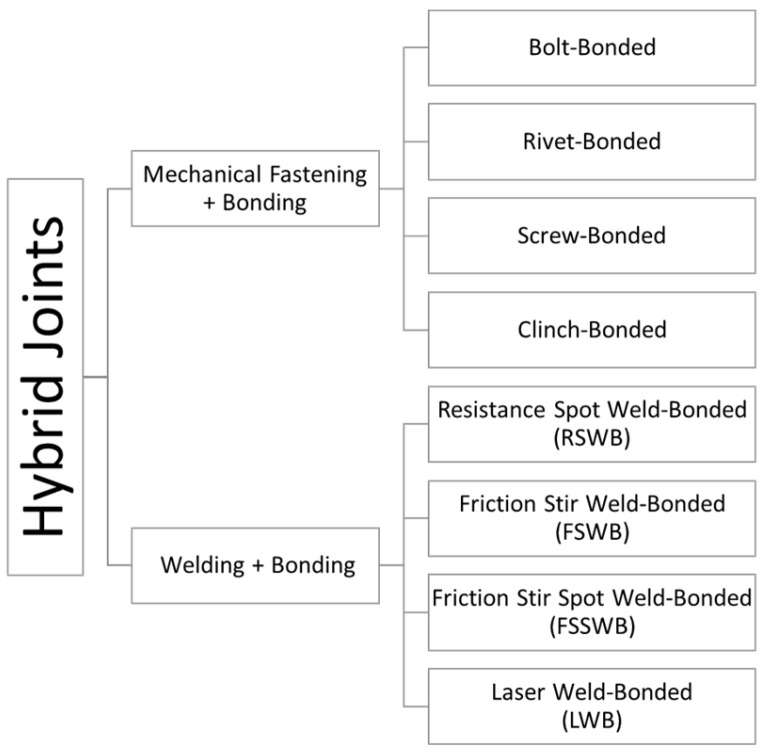
Schematic classification of hybrid joints based on the combination of joining methods, highlighting the weld-bonding techniques considered in this review.

Hybrid joints can be classified according to several criteria, including the type of joining methods used, the load transfer mechanism and the durability of the connection [4,6]. A schematic classification of hybrid joints is presented in Figure 2. Among the various hybrid solutions, configurations such as bonded–fastened, clinch-bonded and weld-bonded joints can be distinguished. While all these types are used in engineering practice, weld-bonded joints are of particular interest due to their ability to combine metallurgical bonding with adhesive load transfer mechanisms [21,23].

Bonded–fastened joints combine mechanical fasteners, such as screws or rivets, with adhesive bonding. In such configurations, the adhesive layer distributes stresses over a larger area, while the fasteners provide additional load-bearing capacity and structural stability [4,5,6,8]. Experimental studies have shown that hybrid screw-bonded and riveted–bonded joints exhibit higher load-bearing capacity and longer fatigue life compared with single-method joints [28,29,30]. In addition, the adhesive layer can act as a sealant, reducing the risk of corrosion and environmental degradation [15,17]. However, the presence of mechanical fasteners may introduce local stress concentrations and increase structural weight, which limits their applicability in highly lightweight structures.

Clinch-bonded joints represent another type of hybrid connection, in which adhesive bonding is combined with local plastic deformation of the sheets to form a mechanical interlock. This solution enables fast and efficient joining without additional fasteners and is widely applied in lightweight structures, particularly in automotive applications. Nevertheless, clinch-bonded joints generally provide lower stiffness and load-bearing capacity than weld-bonded configurations, particularly in high-strength metallic structures [20,30].

Weld-bonded joints are a specific group of hybrid joints that combine welding with adhesive bonding [10,16]. In these joints, the metallic weld provides high local strength, while the adhesive layer improves stress distribution, fatigue performance and corrosion resistance [21,27]. Depending on the welding technique, several variants can be distinguished, including resistance spot weld-bonding (RSWB), friction stir weld-bonding (FSWB), friction stir spot weld-bonding (FSSWB) and laser weld-bonding (LWB) [23,24,26]. These processes differ significantly in terms of heat input, interaction with the adhesive layer and resulting joint performance. Compared with other hybrid joining methods, weld-bonded joints offer a favorable combination of local metallic anchoring and distributed adhesive load transfer. As a result, they may provide higher stiffness, improved fatigue resistance and better structural integrity, especially in thin-walled metallic and multi-material structures [16,21,31]. However, their performance strongly depends on the interaction between welding heat input and adhesive behavior, making process optimization more complex than in conventional welding or adhesive bonding alone [31,32].

Hybrid joints can also be classified according to the load-transfer mechanism between the individual joining methods. In parallel joints, the applied joining techniques operate simultaneously and share the load, which improves damage tolerance and fatigue performance [14,31]. In sequential joints, one joining method acts as the primary load-bearing mechanism, while the secondary method supports the structure under overload or failure conditions [14,27].

Hybrid joints may additionally be divided into detachable and permanent configurations [4,5,6,8]. Detachable joints include mechanically fastened systems, such as bolted or screwed joints combined with adhesive bonding, where disassembly remains possible after removal of the mechanical fastener; however, separation may require partial destruction of the adhesive layer or application of debonding procedures [33,34]. The detachable character of these hybrid systems results primarily from the mechanical fastener, whereas the adhesive layer mainly improves load transfer and stress distribution. Therefore, complete preservation of the adhesive layer after disassembly is generally not expected in service-oriented hybrid configurations. Such configurations are mainly applied in systems requiring maintenance, repair or component replacement [4,13,16].

## 3. Adhesives Used in Hybrid Weld-Bonded Joints

The performance of weld-bonded joints is strongly governed by the interaction between adhesive properties and welding-induced thermal cycles. In hybrid weld-bonding, the adhesive complements the metallic weld by reducing stress concentrations and improving fatigue performance [8,15,21]. During welding, adhesive behavior depends primarily on thermal stability, rheological response and curing conditions. The adhesive layer may be exposed to concentrated thermal cycles, local pressure and, depending on the process type, partial melting or plastic deformation of the surrounding material. These conditions may promote polymer degradation, gas evolution, viscosity changes and bond-line instability, which directly influence weld formation, defect generation and mechanical performance. In addition to thermal effects, environmental exposure and moisture absorption may further affect long-term adhesive durability in weld-bonded structures [18,35,36]. Therefore, the selection of adhesive must consider the welding process, heat input and joint configuration [16,17].

The most commonly used adhesives in weld-bonding include epoxy, polyurethane, acrylic and, in selected cases, thermoplastic systems. These materials differ significantly in terms of thermal resistance, flexibility and behavior under welding conditions, which directly influences their applicability in different weld-bonding processes. Compared with polyurethane and acrylic systems, epoxy adhesives generally provide superior thermal resistance and weld-zone stability during high-heat-input processes, whereas polyurethane and acrylic adhesives offer improved flexibility and damage tolerance under dynamic loading conditions.

A comparative summary of the main properties and application areas of the analyzed adhesive systems is presented in Table 1.

### 3.1. Epoxy Adhesives

Epoxy adhesives are the most commonly used systems in weld-bonding technology due to their high mechanical strength and thermal stability [8,16,41]. They can typically withstand temperatures up to 180–200 °C, making them suitable for high-heat-input processes such as resistance spot weld-bonding (RSWB) and laser weld-bonding (LWB). Under excessive thermal exposure, epoxy systems may undergo softening, gas evolution and local deterioration of cohesive properties, which may promote porosity formation and reduce weld stability [37,38,39,40].

The high cross-link density of epoxy systems contributes to superior joint stiffness and fatigue performance but simultaneously reduces deformability under dynamic loading conditions. In addition, the relatively high viscosity of epoxy systems may influence adhesive flow and local bond-line thickness during welding, affecting stress distribution and weld formation [41,42]. This limited deformability may promote local delamination or crack initiation under dynamic loading conditions. Consequently, epoxy adhesives are particularly suitable for structural weld-bonding applications requiring high stiffness and fatigue resistance, although their relatively brittle behavior may reduce tolerance to peel and impact loading [35,36].

### 3.2. Polyurethane Adhesives

Polyurethane adhesives are characterized by significantly higher flexibility and energy absorption capacity compared with epoxy systems [15,17,34]. This results in improved resistance to dynamic loading, vibration and impact, which is particularly important in thin-walled structures [33,43].

However, their lower thermal resistance limits their application in high-temperature welding processes [12,16]. During processes such as laser welding or high-energy resistance spot welding, thermal degradation of polyurethane adhesives may occur. Under excessive thermal exposure, polyurethane systems may undergo softening, gas evolution and local deterioration of cohesive properties which can promote porosity formation and reduce weld stability [34,40,44,45,46,47]. Consequently, these adhesives are mainly used in low-heat-input processes, such as friction stir-based weld-bonding techniques or modified RSW processes with reduced heat input [12,24,26]. Therefore, polyurethane adhesives are generally more suitable for low- and medium-heat-input weld-bonding processes than for fusion-based techniques involving intense local heating.

### 3.3. Acrylic Adhesives

Acrylic adhesives are used in applications requiring fast curing and good adhesion to metallic surfaces. Compared with epoxy systems, acrylic adhesives generally exhibit improved peel and impact resistance, although their long-term thermal durability remains lower under prolonged welding-related thermal exposure. Their relatively rapid curing behavior may be advantageous for industrial production; however, control of curing conditions remains important to ensure stable adhesive properties during subsequent welding operations [16,48,49]. This may be advantageous in automated manufacturing environments requiring reduced processing time. They exhibit moderate thermal resistance and balanced mechanical properties, making them suitable for selected weld-bonding configurations [35,41]. Acrylic systems generally provide improved resistance to peel and dynamic loading, although their static strength remains lower than that of epoxy adhesives [48,50]. Their moderate thermal stability limits their use in high-heat-input processes, but they can be effectively applied in friction-based welding techniques and in joints where thermal exposure is controlled. Acrylic adhesives are generally more suitable for low- and medium-heat-input weld-bonding processes than for high-energy fusion-based techniques [12,24,26].

### 3.4. Thermoplastic Adhesives

Thermoplastic adhesive systems are characterized by relatively high deformability, good vibration damping capability and the possibility of reprocessing or remelting under elevated temperatures. However, their applicability in weld-bonding technologies remains more limited than that of thermosetting structural adhesives, particularly under high-heat-input conditions [16,17,51].

Unlike thermosetting systems such as epoxies, thermoplastic adhesives do not form a permanently cross-linked network after processing. Consequently, exposure to elevated temperatures during welding may induce local softening, partial melting and changes in bond-line geometry. During fusion-based processes such as resistance spot weld-bonding (RSWB) and laser weld-bonding (LWB), local thermal exposure may lead to adhesive flow, thickness variations, reduction in the effective bonded area and deterioration of load transfer conditions [37,38,39,40,52].

Thermoplastic systems may additionally exhibit higher susceptibility to creep deformation and thermal softening under long-term loading conditions. Nevertheless, their behavior strongly depends on polymer type, molecular architecture and processing conditions. Therefore, thermoplastic adhesives should not be considered universally unsuitable for structural applications, although their use in high-load weld-bonded joints involving elevated process temperatures remains limited compared with thermosetting epoxy systems [17,51].

It should also be noted that classification based solely on polymer family may be ambiguous because some polyurethane systems may exhibit thermoplastic behavior, whereas structural epoxy adhesives remain predominantly thermosetting materials. Consequently, adhesive classification in weld-bonding applications is more appropriately related to curing mechanism and thermal response rather than only chemical family. As a result, thermoplastic adhesives are currently more frequently considered for auxiliary applications, vibration-damping layers, lightweight polymer–metal assemblies and selected low-load configurations, particularly where reduced process temperatures are used and direct thermal interaction between welding and adhesive layers is limited [6,9,52].

### 3.5. Adhesive Behavior Under Welding Conditions

The behavior of structural adhesives during weld-bonding is strongly influenced by the thermal and mechanical conditions generated during the welding process. During welding, the adhesive layer is exposed to concentrated heat input, pressure and, depending on the process type, plastic deformation or partial melting of the surrounding material. These conditions may significantly affect adhesive integrity, rheological behavior and long-term joint performance [16,17,37,38,39,40].

One of the most important phenomena occurring during weld-bonding is thermal degradation of the adhesive layer. In high-heat-input processes, such as resistance spot weld-bonding (RSWB) and laser weld-bonding (LWB), temperatures near the weld zone may exceed the thermal stability limits of the adhesive, leading to polymer decomposition, carbonization and loss of cohesive strength [37,38,39,40,53,54]. Excessive thermal exposure may additionally promote gas evolution and outgassing, resulting in porosity formation, voids and instability of the weld region [40,53,55]. These effects are particularly significant in weld-through configurations, where the adhesive is present directly in the welding zone during weld formation.

Adhesive rheology during welding also plays a critical role in joint formation and stress distribution. Under elevated temperature conditions, viscosity reduction may occur, leading to adhesive flow within the weld region, bond-line thickness variations and redistribution of the adhesive layer around the joint interface [16,17,41]. In resistance spot weld-bonding (RSWB), the adhesive layer is simultaneously exposed to electrode pressure and localized heating, which may promote partial squeeze-out of the adhesive from the overlap region. This phenomenon modifies local bond-line geometry and may affect stress transfer conditions within the joint. The extent of adhesive displacement depends not only on viscosity but also on adhesive curing state, welding pressure, heat input and process sequence. Consequently, optimization of adhesive rheology and process parameters is important for achieving stable weld formation and minimizing local defects in weld-bonded structures [37,38,39,40,53].

Several studies have indicated that adhesive behavior during welding may influence weld nugget formation and, in some cases, modify final nugget dimensions through changes in local heat transfer and adhesive displacement. Changes in adhesive viscosity and squeeze-out intensity may additionally modify local heat transfer conditions and material flow within the weld region, thereby affecting final weld nugget size and morphology. These effects may additionally contribute to defect generation, including voids and porosity associated with gas evolution [37,38,54,56]. In contrast, highly viscous adhesive systems may restrict local material flow and contribute to non-uniform stress distribution within the overlap region.

The curing state of the adhesive prior to welding is another important factor influencing weld-bonding performance. Depending on the process route, welding may be performed before complete adhesive curing or after partial pre-curing of the adhesive layer [21,23,56]. Pre-cured systems generally provide improved dimensional stability during welding, whereas uncured adhesives may exhibit higher susceptibility to local flow, gas formation and thermal degradation [16,37,38,39,40]. As a result, optimization of curing conditions is important for achieving stable weld formation and consistent joint properties.

The influence of welding conditions on adhesive behavior differs significantly between fusion-based and friction-based weld-bonding processes. Fusion-based techniques, such as RSWB and LWB, are generally associated with higher thermal loads and increased risk of adhesive degradation, outgassing and porosity formation [37,38,39,40,53]. In contrast, friction stir weld-bonding (FSWB) and friction stir spot weld-bonding (FSSWB) operate at lower process temperatures and in the solid state, which allows better preservation of adhesive properties and reduces thermal damage [12,24,26,57,58,59,60,61,62]. However, friction-based processes require more precise control of tool movement, local temperature distribution and adhesive thickness and rheological stability to ensure stable joint formation and reproducible mechanical performance.

Overall, adhesive selection in weld-bonding applications requires balancing thermal stability, mechanical performance and process compatibility (Table 1). Thermosetting systems, particularly epoxy adhesives, generally provide superior thermal resistance and load-bearing performance, whereas polyurethane and acrylic systems offer improved flexibility and damage tolerance under dynamic loading conditions. In contrast, thermoplastic systems remain mainly limited to auxiliary or low-load applications due to their lower thermal and structural stability under welding conditions.

The behavior of adhesives during weld-bonding is governed by the complex interaction between thermal exposure, rheological response and welding process conditions. Therefore, optimization of weld-bonding technology requires not only appropriate selection of adhesive systems, but also careful control of heat input, curing conditions and process parameters in order to minimize degradation phenomena and ensure reliable mechanical performance of hybrid joints [16,17,39,53].

## 4. Joining Processes Used in Weld-Bonding

**Figure 3:**
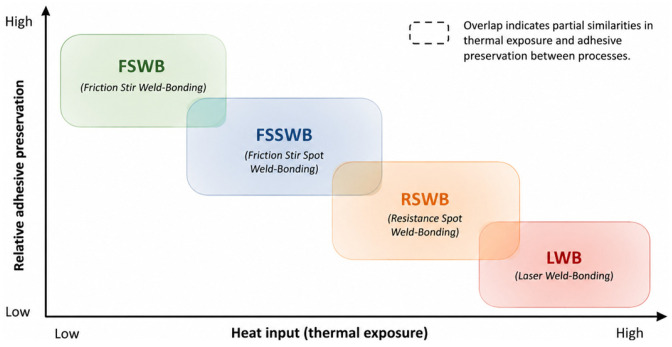
Generalized qualitative relationship between process heat input and relative adhesive preservation in selected weld-bonding technologies.

The welding process is a critical factor governing the performance of weld-bonded joints, as it directly influences microstructural evolution, residual stress distribution and long-term joint durability [6,7,8,9,13,21]. Different welding methods differ substantially in terms of thermal exposure, metallic bonding mechanisms and their interaction with the adhesive layer [10,12,63]. Consequently, adhesive integrity in weld-bonded joints is strongly dependent on the thermal conditions generated during the welding process. A generalized representation of this relationship is presented schematically in Figure 3.

In general, solid-state processes such as FSWB and FSSWB provide better preservation of adhesive properties due to lower thermal exposure, whereas fusion-based processes, including RSWB and LWB, are more susceptible to adhesive degradation, outgassing and local defect formation. However, the relationship between thermal exposure and adhesive degradation should be treated as a generalized trend, since actual adhesive behavior additionally depends on welding time, heat distribution, adhesive chemistry and joint geometry.

From a technological perspective, weld-bonding processes are generally classified into two approaches: the flow-in technique and the weld-through technique [16,23,37]. The fundamental difference between these approaches lies in the interaction between the molten or plasticized material and the adhesive layer, which directly affects stress transfer, defect formation and long-term mechanical performance of the joint [25,27].

**Figure 4:**
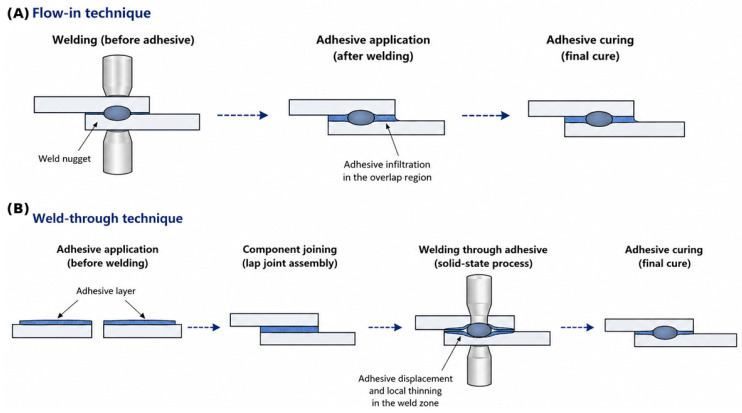
Schematic representation of weld-bonding techniques: (**A**) flow-in technique, where welding is performed prior to adhesive application; (**B**) weld-through technique, where adhesive is applied before welding.

In the flow-in technique (Figure 4A), welding is performed before adhesive application. After weld formation, the adhesive is introduced into the overlap region by capillary penetration, injection or subsequent filling procedures, depending on joint geometry and process configuration [21,39,40,41,42,53]. The adhesive subsequently penetrates the overlap region and contributes to additional load transfer and mechanical anchoring effects. This approach is typically associated with resistance spot weld-bonding (RSWB) and selected laser weld-bonding (LWB) configurations. Because the adhesive is applied after welding, thermal degradation phenomena occurring during weld formation are largely avoided. However, the effectiveness of flow-in configurations strongly depends on adhesive penetration capability, overlap geometry and complete filling of the joint region. Insufficient penetration may result in local void formation, incomplete bonding and non-uniform stress distribution.

In contrast, the weld-through technique (Figure 4B) involves adhesive application before welding. The adhesive is deposited directly between the joined sheets and the welding operation is subsequently performed through the adhesive layer. During welding, the adhesive is exposed to localized thermal and mechanical loading, which may induce adhesive displacement, squeeze-out, local thickness variations and partial adhesive starvation near the weld region [16,17,37,38,39,40]. This approach is characteristic of friction-based processes such as friction stir weld-bonding (FSWB) and friction stir spot weld-bonding (FSSWB) [12,24,57,58,59,60,61,62]. In these systems, preservation of adhesive integrity depends strongly on process temperature, tool movement, material flow and bond-line stability. Although direct mixing between metal and adhesive is generally limited, local interaction between the plasticized metallic zone and displaced adhesive may still affect stress redistribution and final joint morphology.

Consequently, fusion-based processes such as RSWB and LWB promote stronger metallurgical bonding and localized anchoring but simultaneously increase the risk of adhesive degradation and porosity formation [36,37,38,39]. In contrast, solid-state processes including FSWB and FSSWB better preserve adhesive integrity, although their performance depends more strongly on adhesive-assisted load transfer and mechanical interlocking mechanisms [12,24,26].

A comparative overview of the principal weld-bonding processes, including thermal conditions, adhesive preservation, characteristic defects and structural performance, is presented in Table 2.

### 4.1. Resistance Spot Weld-Bonding (RSWB)

Resistance spot weld-bonding (RSWB) is the most widely used hybrid joining method, particularly in the automotive industry, due to its high productivity and compatibility with automated manufacturing systems. In this process, electrodes apply pressure and conduct electric current, resulting in localized fusion, rapid solidification and formation of a weld nugget [7,10,13,14,31,32,37,38,39,52,63].

Depending on the process sequence, RSWB can be implemented using both the flow-in and weld-through techniques. In the flow-in approach, welding is performed prior to adhesive application, and the adhesive is subsequently introduced into the joint region, where it penetrates the overlap and enhances stress redistribution and local load transfer within the overlap region [21,37,38,39,53]. In contrast, in the weld-through approach, the adhesive is applied before welding, and the weld is formed through the adhesive layer while maintaining separation between the adhesive layer and molten metal [37,38,39,40].

The presence and sequence of the adhesive layer significantly influence current flow, local thermal gradients, cooling rate and stress distribution within the joint [14,21,23,27,31,32,64,65,66,67,68]. In the flow-in configuration, the adhesive primarily contributes to stress redistribution and sealing after weld formation, whereas in the weld-through configuration it directly interacts with the welding process, which may lead to local degradation, outgassing and void formation [27,37,38,39,54,56,64,65,66,67,68,69].

Despite these differences, both approaches improve static load-bearing capacity and fatigue resistance compared with conventional spot welding. However, weld-through configurations are generally more sensitive to process parameters and adhesive properties, while flow-in configurations provide more stable welding conditions at the expense of reduced interaction between the weld and adhesive layer. These limitations originate mainly from the simultaneous action of high temperature and electrode pressure, which directly affects adhesive integrity. Therefore, practical implementation of RSWB requires careful optimization of welding current, electrode force and adhesive thermal resistance in order to minimize degradation and defect formation [27,37,38,39,64].

### 4.2. Friction Stir Weld-Bonding (FSWB)

In friction stir weld-bonding (FSWB), the joint is formed in the solid state through severe plastic deformation and frictional heating generated by a rotating tool. The process temperature is significantly lower than in fusion welding, which allows the adhesive to retain much of its original mechanical and rheological performance [11,12,24,26,59,60,61].

FSWB corresponds to the weld-through approach, in which the adhesive is applied prior to welding and the rotating tool passes through the adhesive layer while generally maintaining separation between the adhesive and metallic phases. As a result, a continuous joint is produced with low residual stresses, high fatigue resistance and limited microstructural degradation. In addition, the reduced thermal gradients generated during FSWB contribute to lower residual stress development compared with fusion-based weld-bonding techniques. Compared with fusion-based processes, FSWB offers better preservation of adhesive properties and reduced thermal degradation. However, it requires precise control of process parameters and is currently most widely applied to aluminum alloys and lightweight metallic structures. The main technological challenge in FSWB is maintaining stable material flow while preserving adhesive continuity and bond-line stability during tool movement. Consequently, industrial implementation requires precise control of rotational speed, axial force and process temperature to avoid local bond-line disturbances [12,24,25,26,57,58,59,60,61,64].

### 4.3. Friction Stir Spot Weld-Bonding (FSSWB)

Friction stir spot weld-bonding (FSSWB) is a localized variant of FSW, used where it is necessary to limit heat input or to join materials with reduced weldability. The rotating tool locally plasticizes the material without melting, thereby reducing thermal degradation of the adhesive layer [12,24,57,58,59,60,61,62,66,70,71].

Similarly to FSWB, this process follows the weld-through mechanism, in which the adhesive is applied prior to welding and the rotating tool passes through the adhesive layer without significant mixing with the metal. This ensures relatively stable adhesive behavior and limited thermal degradation. FSSWB joints exhibit more favorable local stress distribution and improved resistance to interfacial delamination, particularly in thin-walled structures. However, compared with continuous FSWB joints, FSSWB joints generally exhibit lower load-bearing capacity due to their localized nature. This limitation results primarily from the discontinuous character of spot joints and reduced effective load transfer area compared with continuous weld-bonded configurations. Nevertheless, FSSWB remains attractive for thin-walled and lightweight structures where minimization of heat input is critical [12,24,25,26,27,57,58,59,60,61,62,64,66,70,71,72,73].

### 4.4. Laser Weld-Bonding (LWB)

Laser weld-bonding (LWB) enables the production of high-precision joints with a narrow heat-affected zone, steep local thermal gradients and minimal global distortion [53,74,75,76,77,78,79,80,81,82]. The highly concentrated laser beam induces rapid localized fusion and solidification of the base material, which significantly influences the interaction between the weld and the adhesive layer.

Depending on the process sequence, LWB can be implemented using both flow-in and weld-through approaches. In the flow-in configuration, welding is performed prior to adhesive application, and the adhesive subsequently penetrates the joint region, enhancing mechanical anchoring. In contrast, in the weld-through configuration, the adhesive is applied before welding, and the laser beam passes through the adhesive layer without significant mixing with the metal [37,38,39,40,74,75,76,77,78,79,80].

Due to the high energy density of the laser beam, LWB promotes steep thermal gradients, which may lead to adhesive degradation, gas evolution and the formation of defects such as porosity or voids. These phenomena may additionally contribute to residual stress accumulation and reduced long-term fatigue performance of the joint. Therefore, optimization of welding parameters and appropriate selection of thermally resistant adhesive systems are essential for achieving stable joint quality. Compared with resistance spot weld-bonding (RSWB), LWB offers higher precision and better dimensional control, although it requires stricter optimization of processing conditions and selection of thermally stable adhesive systems. The dominant limitations of LWB arise from highly concentrated heat input and steep thermal gradients, which intensify adhesive degradation and gas evolution phenomena. Therefore, practical implementation requires strict optimization of laser power, welding speed and adhesive thermal resistance [37,38,39,53,55,74,75,76,77,78,79]. From an application perspective, fusion-based weld-bonding processes are generally preferred in high-productivity manufacturing environments requiring strong local metallurgical bonding, whereas friction-based techniques are more suitable for lightweight structures where preservation of adhesive integrity and minimization of thermal distortion are critical.

## 5. Mechanical Performance of Weld-Bonded Joints

The mechanical performance of weld-bonded joints is governed by the interaction between the adhesive layer, the metallic weld and the resulting stress transfer mechanisms within the hybrid joint structure. Compared with conventional welded or adhesively bonded joints, weld-bonded configurations may provide improved load distribution, delayed crack initiation and enhanced fatigue resistance. However, these improvements strongly depend on welding parameters, adhesive properties, bond-line thickness, joint geometry and the degree of adhesive degradation during welding [14,22,23,25,27,31,32].

**Figure 5:**
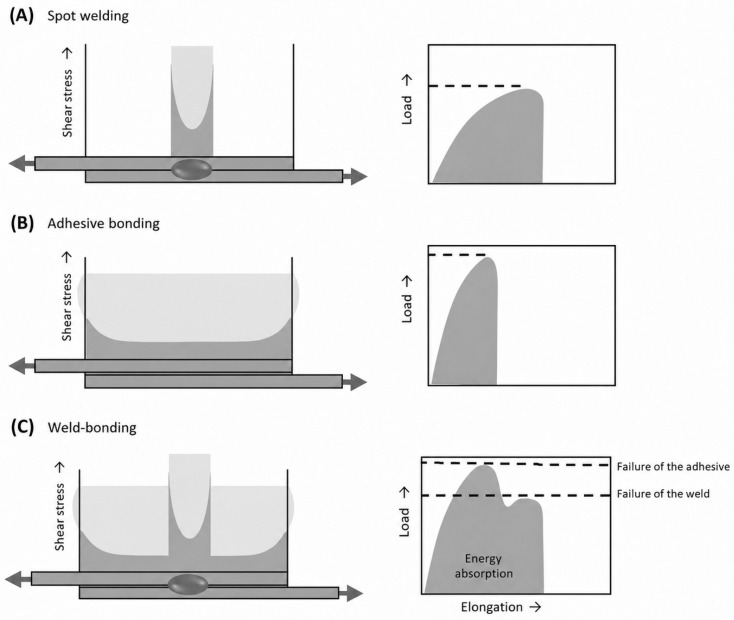
Schematic comparison of stress distribution and representative load–displacement behavior in (**A**) spot-welded, (**B**) adhesively bonded and (**C**) weld-bonded joints, illustrating differences in load transfer and stress redistribution mechanisms.

The fundamental differences in stress distribution and load–displacement behavior between welded, adhesively bonded and weld-bonded joints are illustrated schematically in Figure 5. A comparative overview of the mechanical response, fatigue behavior and dominant failure characteristics of major weld-bonding processes is presented in Table 3.

### 5.1. Static Strength

The static load-bearing capacity of weld-bonded joints depends on weld size, adhesive thickness, overlap geometry, curing conditions, process parameters and adhesive properties. The presence of the adhesive layer redistributes stresses over a larger overlap area, reducing global stress concentration within the overlap region and delaying crack initiation [25,27,56,64,84,85,90,91]. However, stress redistribution in weld-bonded joints is not completely uniform. Local stress concentration may still develop near the transition region between the weld nugget and the adhesive layer due to the abrupt change in stiffness and load transfer mechanism. Numerical and experimental investigations have shown that crack initiation frequently remains associated with the weld region, particularly under cyclic loading conditions and in joints affected by residual stresses or weld-related defects [67,69,82,84,85].

Stress concentrations developing near the transition region between the weld nugget and the adhesive layer may additionally lead to the formation of local stress singularities associated with abrupt changes in geometry, stiffness and load transfer mechanisms. These singular regions are typically located at the weld edge, overlap ends and weld–adhesive interfaces, where tensile, shear and peel stresses may locally increase. This effect becomes particularly pronounced at interface edges, where local stress amplification may accelerate crack initiation and promote mixed adhesive–metal failure. The presence of stress singularities may promote crack initiation, interfacial failure and premature damage accumulation under cyclic loading conditions. Their intensity strongly depends on adhesive thickness, local bond-line geometry and stiffness mismatch between the metallic weld and the adhesive layer. Increasing adhesive thickness may partially reduce local stress peaks through improved stress redistribution; however, excessive bond-line thickness may simultaneously introduce local instability, adhesive defects and non-uniform load transfer. Therefore, optimization of adhesive geometry and interface design remains important for minimizing stress singularities and improving fatigue performance of weld-bonded structures [64,67,82,84,85].

Adhesive thickness additionally influences local mechanical response. Thin adhesive layers generally increase joint stiffness and improve load transfer efficiency but may simultaneously promote higher local stress concentration near the weld region. In contrast, thicker bond lines enable more gradual stress redistribution and improved energy dissipation. However, excessive adhesive thickness may contribute to bond-line instability, local void formation and variations in stress transfer, especially when adhesive displacement occurs during welding [66,69,72,73].

Although the adhesive layer generally improves mechanical response, excessive adhesive flow, thermal degradation or gas evolution during welding may promote defect formation and reduce weld stability [37,38,39,40,53,55]. In flow-in configurations, partial penetration of the adhesive into the overlap region contributes to improved initial stiffness and enhanced load sharing between the weld and adhesive layer. In contrast, weld-through configurations rely more strongly on preservation of adhesive integrity and distributed stress transfer during welding.

The magnitude of static strength improvement depends strongly on the welding process and joint configuration. Resistance spot weld-bonding (RSWB) joints generally exhibit high local stiffness and load-bearing capacity due to the formation of a distinct weld nugget and strong metallurgical anchoring. Experimental investigations performed for dual-phase steels demonstrated tensile–shear strength increases of approximately 40% for DP590 steel and 58% for DP780 steel relative to conventional spot-welded joints. Additional increases of approximately 15% and 39%, respectively, were reported compared with adhesive-bonded configurations [83].

Friction stir weld-bonding (FSWB) joints provide more uniform stress transfer along the overlap and reduced local stress concentration. For AA6082-T6 systems, average joint efficiencies of approximately 73.8% and maximum values up to approximately 85.2% relative to the base material have been reported [86]. In friction stir spot weld-bonding (FSSWB), the localized nature of the process generally results in lower load-bearing capacity compared with continuous FSWB joints; however, hybrid configurations still demonstrated considerable mechanical improvement. Although FSSWB joints generally exhibit lower load-bearing capacity than continuous FSWB configurations, the reduction is frequently compensated for by lower heat input, reduced distortion and improved applicability to thin-walled structures. Tensile–shear failure load increased by approximately 2.7 times and joint stiffness by approximately 1.1 times compared with conventional FSSW joints [66].

Laser weld-bonding (LWB) combines localized metallurgical bonding with high dimensional precision and concentrated load transfer. Representative laser joining systems demonstrated tensile–shear loads up to approximately 5.6 kN and strength improvements of about 25% relative to reference laser configurations [87]. Nevertheless, mechanical performance remains strongly dependent on adhesive integrity because adhesive degradation, gas evolution and porosity formation may locally reduce the effective bonded area and modify stress transfer mechanisms [40,53,55].

Overall, reported improvements in static load-bearing capacity vary considerably depending on material combination, adhesive system, weld geometry and test configuration. Although weld-bonded joints frequently exhibit higher static strength than conventional welded joints, increased load-bearing capacity does not necessarily correspond to proportional improvement in fatigue resistance. In fusion-based weld-bonded joints, high local stiffness and strong metallurgical anchoring may coexist with residual tensile stresses and local stress concentration near the weld region, which may accelerate fatigue crack initiation under cyclic loading conditions [31,64,68].

### 5.2. Fatigue Performance

Fatigue resistance is a critical parameter for the application of weld-bonding in transport and engineering structures. The adhesive layer reduces stress concentration in the weld zone, dampens vibrations and delays crack initiation, leading to improved fatigue life compared with conventional welded joints [22,23,25,27,84,85,90,91]. Nevertheless, fatigue improvement is not universal. In some configurations, thermally induced adhesive degradation, void formation, residual stresses or nugget defects may reduce the expected benefit of hybridization and shift crack initiation back toward the weld zone.

Although numerous studies report improved fatigue performance of weld-bonded joints due to stress redistribution and delayed crack initiation, contradictory findings have also been reported in the literature. In some configurations, the beneficial effect of the adhesive layer may be partially reduced by welding-induced residual stresses, local adhesive degradation, porosity formation or interfacial defects generated during the welding process [40,53,54,56,64,65,66,67,68,69]. Studies on hybrid welded structures have shown that residual tensile stresses may accelerate fatigue crack propagation and reduce the expected improvement in fatigue life [16,31,64]. Therefore, the fatigue response of weld-bonded joints should be considered strongly dependent on process optimization, adhesive integrity and local stress distribution rather than as an inherent advantage of hybrid joining in all configurations.

The fatigue behavior differs considerably between weld-bonding technologies. In weld-through processes such as friction stir weld-bonding (FSWB) and friction stir spot weld-bonding (FSSWB), the relatively low process temperature allows preservation of a significant portion of the adhesive elastic and damping properties, contributing to improved cyclic performance. Quantitative investigations on AA6082-T6 joints showed that fatigue strength at 10^6^ cycles reached approximately 79.9% of the adhesive-bonded joint strength for FSWB configurations, whereas overlap FSW joints reached only approximately 41.6% [26]. These results indicate that hybridization may nearly double the retained fatigue performance relative to friction stir overlap joints without adhesive support.

For FSSWB configurations, hybrid joints additionally demonstrated improved resistance to interfacial crack propagation and cyclic damage accumulation. Failure energy values approximately 8.14 times higher than those of conventional FSSW joints were reported, indicating a substantially improved ability to dissipate energy during progressive damage development [66].

In resistance spot weld-bonding (RSWB), the weld nugget provides localized load transfer and high local stiffness; however, residual tensile stresses and stiffness mismatch between the weld region and adhesive layer may promote crack initiation near the weld nugget under cyclic loading conditions [31,40,53,54,56,64,65,66,67,68,69]. Experimental studies on dual-phase steels reported improved endurance limits at approximately 10^6^ cycles compared with conventional spot-welded joints, although fatigue response remained sensitive to nugget defects and local adhesive degradation [83].

Laser weld-bonded (LWB) joints generally exhibit stronger dependence on process optimization because concentrated heat input may promote adhesive degradation, porosity formation and local interfacial defects [40,53,77,78,79,80]. Comparative studies indicated that adhesive-bonded joints may exhibit fatigue strength up to approximately five times higher than laser-welded joints, whereas weld-bonded configurations showed intermediate fatigue behavior [89]. This suggests that preservation of adhesive integrity remains a critical factor controlling long-term cyclic performance in laser-assisted hybrid joints.

From a comparative perspective, FSWB and FSSWB joints frequently exhibit more favorable fatigue behavior than fusion-based weld-bonding technologies because of lower thermal gradients, reduced residual stresses and improved preservation of adhesive properties. In contrast, RSWB and LWB systems generally provide higher local stiffness and stronger metallurgical anchoring but simultaneously remain more sensitive to thermally induced defects and stress concentration effects.

Overall, the fatigue performance of weld-bonded joints is governed by the interaction between stress redistribution, residual stresses, adhesive degradation and crack propagation mechanisms. Consequently, fatigue response should be interpreted as a process–adhesive–geometry-dependent phenomenon rather than as an inherent advantage of weld-bonding in all configurations.

### 5.3. Energy Absorption and Impact Performance

Weld-bonded joints exhibit favorable energy absorption characteristics due to the combined action of the deformable adhesive layer and the stiff metallic weld. The mechanisms responsible for energy absorption differ between the metallic and adhesive components of the hybrid structure. The metallic weld primarily dissipates energy through elastic–plastic deformation, local yielding and crack propagation within the joined sheets. In contrast, the adhesive layer contributes mainly through viscoelastic deformation, interfacial debonding processes and vibration damping, resulting in more gradual stress redistribution under dynamic loading conditions [25,30,68,72].

Consequently, the overall energy dissipation capacity of weld-bonded joints results from the combined interaction of metallic plasticity and adhesive-assisted deformation mechanisms rather than from the weld alone. This distinction becomes particularly important in lightweight structures subjected to crash loading or impact conditions, where adhesive integrity may significantly influence deformation mode, failure sequence and local damage evolution.

Specific energy absorption (SEA) is increasingly considered an important parameter for evaluating crash-related performance of hybrid joints. Although reported values vary considerably depending on material system, overlap geometry and loading conditions, several studies indicate that weld-bonded configurations may provide improved energy absorption compared with purely welded joints when adhesive integrity is preserved during welding and subsequent loading [30,31,68]. Experimental investigations on hybrid FSSWB configurations demonstrated approximately 8.14-fold higher failure energy compared with conventional FSSW joints, indicating a substantial increase in the ability to dissipate energy during progressive damage development. In addition, numerical analyses showed a reduction in local peak stress from approximately 243.1 MPa to 15.5 MPa under a loading force of 2 kN, suggesting more effective stress redistribution and delayed damage localization [66].

Under dynamic loading, the adhesive layer additionally promotes stress redistribution and delays localized failure development. In flow-in configurations, partial penetration of the adhesive into the overlap region contributes to improved initial stiffness and enhanced load sharing. In contrast, weld-through configurations rely more strongly on adhesive deformability and preservation of interfacial integrity during loading.

From a process perspective, friction-based techniques such as FSWB and FSSWB may provide favorable impact performance because lower thermal exposure allows better preservation of adhesive properties. Fatigue investigations additionally showed that FSWB joints retained approximately 79.9% of the fatigue strength of adhesive-bonded joints at 10^6^ cycles, whereas overlap FSW joints retained only approximately 41.6%, indicating improved damage tolerance and more efficient energy dissipation under cyclic loading conditions [26].

In comparison, RSWB joints generally exhibit higher local stiffness but lower ability to accommodate extensive plastic deformation under dynamic loading because energy absorption is dominated mainly by localized metallic deformation near the weld nugget. Laser weld-bonded joints may provide high dimensional precision; however, their impact response remains sensitive to adhesive degradation and porosity formation generated by concentrated heat input [40,53,55,77,78,79,80].

Overall, energy absorption in weld-bonded joints should be considered a combined result of metallic plasticity, adhesive viscoelastic deformation and interfacial damage evolution. Therefore, optimization of impact performance requires simultaneous control of weld geometry, adhesive properties, bond-line thickness and thermal effects generated during joining.

### 5.4. Failure Modes

The failure mechanisms of weld-bonded joints depend on the interaction between the welding process and the adhesive layer. Common failure modes include adhesive failure at the interface, cohesive failure within the adhesive, fracture within the metallic region and mixed-mode failure. The transition between adhesive, cohesive and mixed-mode failure is strongly affected by local thermal degradation, interfacial adhesion, residual stress distribution and weld geometry [22,23,25,27,84,85,90,91].

In flow-in configurations, partial interaction between the weld and adhesive promotes mixed-mode failure, with the fracture path alternating between the adhesive layer and the metallic region. In contrast, weld-through processes such as friction stir weld-bonding (FSWB) and friction stir spot weld-bonding (FSSWB) tend to exhibit more distributed damage, including cracking within the plasticized zone and progressive failure within the adhesive layer.

From a comparative perspective, resistance spot weld-bonding (RSWB) joints are more prone to crack initiation near the weld nugget because local metallurgical anchoring generates high stiffness gradients between the weld region and the surrounding adhesive layer. Experimental studies reported crack initiation and mixed adhesive–metal failure near the nugget region under cyclic loading conditions [67,69,82]. Although RSWB provides high local load-bearing capacity, residual tensile stresses and nugget defects may accelerate crack propagation and reduce fatigue performance [31,64,68].

In friction stir weld-bonding (FSWB), lower process temperatures and reduced residual stresses generally promote more uniform damage distribution. Fatigue investigations showed that FSWB joints retained approximately 79.9% of the fatigue strength of adhesive-bonded joints at 10^6^ cycles, whereas overlap FSW joints retained only approximately 41.6% [26]. This behavior suggests delayed crack localization and more distributed failure development within the adhesive layer and stir zone.

Hybrid FSSWB configurations exhibited a pronounced shift in damage evolution compared with conventional FSSW joints. Crack propagation was transferred from localized weld-edge regions toward the adhesive layer, accompanied by an approximately 8.14-fold increase in failure energy and reduction in local peak stress from approximately 243.1 MPa to 15.5 MPa under 2 kN loading [66]. These results indicate that adhesive participation significantly modifies local stress concentration and failure sequence.

In laser weld-bonded (LWB) joints, failure behavior remains strongly dependent on process parameters and adhesive thermal stability. Excessive heat input may promote adhesive decomposition, gas evolution and porosity formation, leading to interfacial defects and localized fracture near the weld region [40,53,55]. Compared with friction-based techniques, LWB joints generally exhibit stronger sensitivity to thermal gradients and local degradation phenomena.

Overall, the observed failure mechanisms indicate that the mechanical response of weld-bonded joints cannot be attributed solely to the nominal welding process. Instead, failure behavior results from the combined effect of adhesive integrity, weld quality, residual stresses, local stress redistribution and thermally induced degradation phenomena.

Consequently, the mechanical response of weld-bonded joints depends not only on the welding process but also on adhesive family and substrate material. High-stiffness epoxy systems generally favor load transfer and fatigue resistance, whereas polyurethane systems promote energy absorption and damage tolerance. Acrylic adhesives provide balanced stiffness and flexibility together with improved dynamic performance. In addition, substrate selection influences process applicability, because aluminum alloys are frequently associated with friction-based techniques, while composite–metal systems require additional consideration of interfacial compatibility and thermal mismatch.

## 6. Applications of Weld-Bonded Joints

Weld-bonded joints are increasingly applied in engineering structures requiring a combination of high load-bearing capacity, fatigue resistance, sealing performance and weight reduction. Their industrial relevance is particularly significant in lightweight multi-material structures, where conventional welding or adhesive bonding alone may not provide sufficient structural efficiency, durability or damage tolerance [1,2,3,4,5,6,13,14,16,21,92,93]. From an application perspective, the main advantage of weld-bonding lies not only in improved joint strength but also in the possibility of combining localized metallurgical anchoring with more uniform stress distribution provided by the adhesive layer. However, the industrial implementation of weld-bonding technologies remains strongly dependent on process compatibility, adhesive thermal stability, production efficiency and the ability to ensure reliable long-term joint durability under service conditions.

### 6.1. Automotive Applications

The automotive industry currently represents the most mature and industrially developed field of application for weld-bonded joints. This is primarily associated with the widespread use of thin steel sheets, aluminum alloys and multi-material body structures, where joining technologies must simultaneously provide high production efficiency, fatigue resistance, dimensional stability and crashworthiness [2,3,9,10,13,31,88]. Resistance spot weld-bonding (RSWB) remains particularly attractive for body-in-white structures because it is compatible with automated manufacturing lines and can often be integrated with existing resistance spot welding infrastructure without major modifications. However, large-scale automotive implementation of weld-bonding technologies still requires balancing production speed, adhesive curing conditions, corrosion protection and long-term durability under cyclic service loading [31,32,37,38,39].

**Figure 6:**
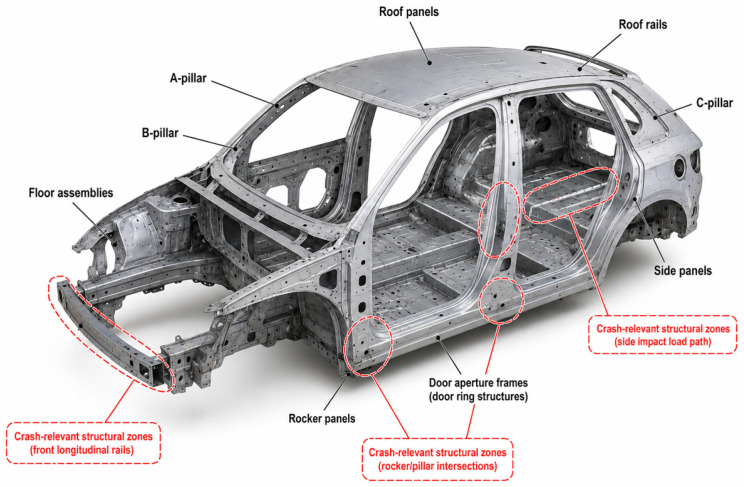
Representative body-in-white (BIW) regions where weld-bonding technologies are commonly applied in automotive structures.

In automotive body-in-white (BIW) structures, weld-bonding is applied in roof panels, floor assemblies, side panels, rocker panels, A-, B- and C-pillars, door frames, roof rails, reinforcing beams and crash-relevant structural zones (Figure 6) [2,3,9,13,94,95,96]. These regions require high structural stiffness, dimensional stability and fatigue resistance while maintaining low vehicle weight and effective crash-energy absorption. In multi-material BIW structures, weld-bonding is additionally applied in aluminum–steel assemblies and lightweight reinforcement regions, where the adhesive layer contributes to stress redistribution, vibration damping and corrosion protection, while the weld provides localized structural anchoring and load transfer [16,31,94,95,96].

In electric vehicle (EV) structures, the importance of weld-bonding is increasing due to the widespread use of aluminum battery enclosures, thin-walled profiles and multi-material assemblies. Typical applications in electric vehicles include aluminum battery housings, battery trays, cooling plate assemblies and thin-walled extruded structural profiles, where minimization of thermal distortion and preservation of dimensional accuracy are particularly important. In such applications, friction-based techniques including FSSWB and refill friction stir spot welding (RFSSW) are particularly attractive because they minimize thermal distortion and reduce the risk of damage to thermally sensitive battery components [11,12,57,58,59,60,61,62]. Compared with RSWB, friction-based weld-bonding processes are generally less thermally aggressive and provide better preservation of adhesive integrity. However, their broader industrial implementation in high-volume automotive manufacturing remains limited by tool wear, process speed and the complexity of controlling adhesive flow and bond-line stability during joining.

### 6.2. Aerospace Applications

**Figure 7:**
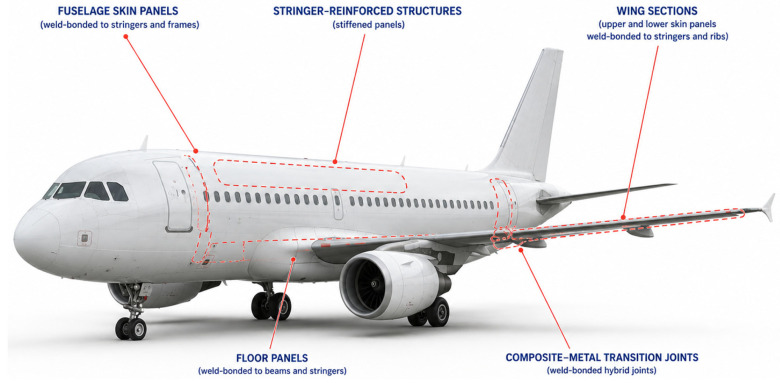
Representative aerospace structural regions where weld-bonding technologies are applied.

In aerospace structures, weld-bonding is primarily considered for applications requiring high specific strength, fatigue resistance and dimensional stability under cyclic loading conditions. Historically, hybrid joining concepts in aerospace structures were introduced to reduce the number of mechanical fasteners, improve structural stiffness and decrease local stress concentrations around riveted regions [21,23,90]. Current aerospace applications focus mainly on aluminum alloys, lightweight stiffened panels, composite–metal assemblies and lightweight thin-walled structures subjected to cyclic loading. Representative applications include fuselage skin panels, stringer-reinforced structures, floor panels, wing sections and lightweight composite–metal transition joints, where a reduction in structural weight and improvement of fatigue resistance are particularly important (Figure 7) [5,11,24,26,64,92,97].

Composite–metal transition joints represent a particularly challenging application area for weld-bonding technologies in aerospace structures. Typical material combinations include CFRP–aluminum and CFRP–titanium systems used in lightweight fuselage components, stiffened panels and transition regions between composite skins and metallic substructures. In such joints, the adhesive layer plays an important role in reducing local stress concentration, accommodating differences in stiffness and partially compensating for the mismatch in thermal expansion between composite and metallic components. However, these systems remain susceptible to interfacial delamination, galvanic corrosion in carbon fiber–metal interfaces, residual stress development and local damage accumulation under cyclic loading conditions [21,23,83,97]. Consequently, reliable implementation of weld-bonding technologies in composite–metal aerospace structures requires careful optimization of adhesive selection, interfacial design and environmental durability.

Friction stir weld-bonding (FSWB) is particularly attractive for aerospace aluminum structures because the solid-state nature of the process minimizes melting-related defects, reduces thermal distortion and limits residual stress development compared with fusion-based welding [11,12,26,64,70,71]. In such configurations, the adhesive layer contributes to stress redistribution and damping of local stress concentrations, whereas the solid-state weld ensures structural continuity and local load transfer. In contrast, laser weld-bonding (LWB) may provide high dimensional precision and limited global distortion; however, the high local thermal gradients generated during laser processing require strict control of heat input and adhesive thermal stability in order to minimize degradation and porosity formation [53,55,74,75,76,77,78,79,80].

However, the implementation of weld-bonding technologies in aerospace structures remains strongly constrained by certification requirements, long-term durability validation and the need for reliable non-destructive inspection of hybrid joints. Consequently, industrial application is currently more advanced in secondary lightweight structures than in primary safety-critical aircraft components.

Overall, aerospace applications favor weld-bonding processes capable of minimizing thermal damage, residual stresses and geometric distortion while maintaining reliable fatigue performance under cyclic service conditions. Consequently, friction-based weld-bonding techniques are generally considered more suitable for fatigue-sensitive lightweight aluminum structures, whereas laser-based solutions may be advantageous for precision components requiring high dimensional accuracy and localized heat input control.

### 6.3. Railway and Heavy Industrial Applications

**Figure 8:**
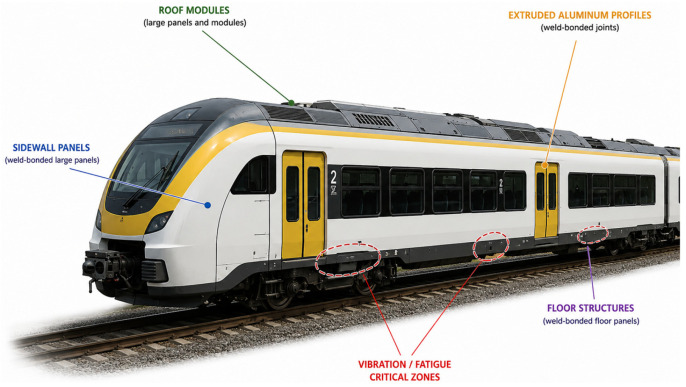
Representative railway and heavy industrial structural regions where weld-bonding technologies are applied.

In railway vehicles and heavy industrial structures, weld-bonded joints are primarily applied in large structural panels, extruded aluminum profiles, cabin modules and components subjected to cyclic loading and vibration [5,6,98,99,100]. The main advantage of weld-bonding in such applications is the possibility of reducing the number of mechanical fasteners or conventional welds while improving structural stiffness, sealing performance and fatigue resistance under long-term service loading. Representative applications include railway sidewall panels, floor structures, roof modules, aluminum carriage sections, cabin structures and lightweight support panels exposed to cyclic service loading and dynamic operating conditions (Figure 8).

For railway applications, friction-based weld-bonding processes are particularly attractive for joining large aluminum profiles and thin-walled structures because they reduce thermal distortion and residual stress development in large structural assemblies [11,12,26,57,58,64,98]. In heavy industrial equipment, weld-bonding may be applied in load-bearing frames, protective housings, structural panels and protective enclosures exposed to cyclic loading and vibration, where the adhesive layer improves stress redistribution while the weld provides localized structural reinforcement [6,28,30]. In railway structures, additional challenges include long-term exposure to vibration, humidity, temperature fluctuations and corrosive environments, all of which may influence long-term adhesive durability and interfacial stability during service. However, industrial implementation of weld-bonding technologies in railway and heavy engineering sectors remains more demanding than in automotive manufacturing. Large structural dimensions, variable service conditions and the need for reliable inspection and certification require robust process control, standardized design methodologies and advanced non-destructive evaluation techniques [98,99,100,101,102,103,104].

### 6.4. Other Industrial Applications

Beyond transportation sectors, weld-bonding technologies are also applied or investigated in household appliances, energy storage systems, electronic assemblies, battery modules and industrial enclosure structures [15,16,74,75,76,79,80,93,105]. In such applications, the main advantages of weld-bonding include improved sealing performance, enhanced vibration damping, corrosion resistance and the possibility of joining materials with dissimilar thicknesses, stiffnesses or thermal properties. Representative applications include battery enclosures, cooling plate assemblies, electronic casings, lightweight appliance panels and protective industrial housings requiring dimensional stability and vibration resistance during operation.

In battery systems and electronic assemblies, laser-based and friction-based weld-bonding processes are particularly attractive because they provide high dimensional precision and relatively localized thermal influence on surrounding components [74,75,76,77,78,79,80,105]. However, thermally induced adhesive degradation, local outgassing and interfacial instability remain critical limitations in processes involving concentrated heat input or thermally sensitive components. Therefore, optimization of adhesive selection, thermal stability and processing parameters is essential for achieving reliable long-term joint performance.

The integration of weld-bonded structures with advanced non-destructive testing (NDT) and structural health monitoring (SHM) systems may further support their application in safety-critical sectors, including energy infrastructure, transportation systems and industrial equipment [102,103,104,106,107]. Nevertheless, broader industrial implementation still requires improved predictive models for long-term durability, clearer design methodologies, standardized inspection procedures and better understanding of environmentally assisted degradation phenomena under variable service conditions.

## 7. Challenges, Limitations, and Future Trends in Weld-Bonded Joints

The mechanical performance and industrial applicability of weld-bonded joints cannot be interpreted solely as a function of the welding process. Adhesive family, substrate material and process conditions interact simultaneously and jointly determine stress redistribution, fatigue behavior and failure evolution. Structural epoxy systems generally provide high stiffness and load-bearing capacity, whereas polyurethane adhesives favor energy absorption and damage tolerance. Thermoplastic systems may offer improved deformability but remain more sensitive to thermal softening. Similarly, substrate selection influences both adhesive compatibility and process applicability. Aluminum alloys are frequently associated with friction-based processes because of reduced thermal distortion, whereas multi-material and composite–metal assemblies require additional consideration of stiffness mismatch, thermal expansion differences and interfacial durability. Despite their advantages, weld-bonded joints still face several technological, material and design-related challenges. Their performance depends on the interaction between welding parameters, adhesive behavior and joint geometry. Consequently, reliable application of weld-bonding requires precise control of heat input, adhesive selection, process stability, quality inspection and structural design procedures [6,16,23,72,90].

**Figure 9:**
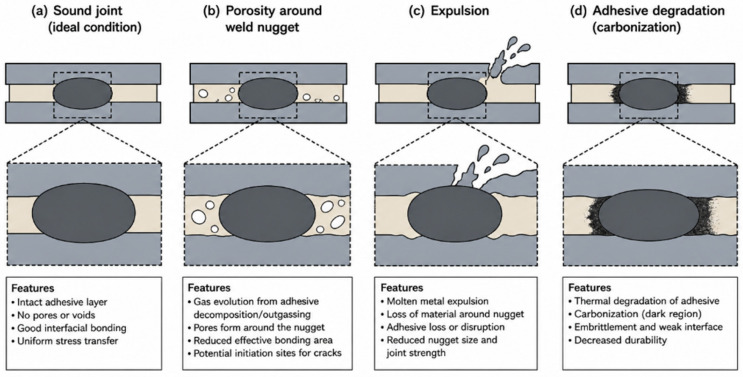
Schematic illustration of typical defect mechanisms in fusion-based weld-bonded joints: (**a**) sound joint with intact adhesive layer; (**b**) porosity formation caused by adhesive decomposition and gas evolution around the weld nugget; (**c**) expulsion resulting from excessive heat input and material loss; and (**d**) thermal degradation of the adhesive manifested by carbonization and interfacial weakening.

One of the major limitations is the thermal interaction between the welding process and the adhesive layer. In high-heat-input processes such as resistance spot weld-bonding (RSWB) and laser weld-bonding (LWB), localized heating may induce adhesive degradation, outgassing, porosity formation and changes in adhesive rheology [37,38,39,40,53,54]. Typical defect mechanisms occurring in fusion-based weld-bonded joints are schematically presented in Figure 9. Thermal decomposition and gas evolution may reduce the effective bonded area, promote local defects and decrease both static and fatigue performance. In addition, excessive heat input may lead to adhesive squeeze-out, local adhesive starvation and changes in bond-line thickness, which further modify load transfer conditions and weld nugget development.

In contrast, friction-based weld-through processes such as FSWB and FSSWB operate at lower process temperatures and generally provide improved preservation of adhesive integrity [12,26,57,58,59,60,61,62,66,71]. However, these processes remain highly sensitive to tool parameters, local material flow, adhesive displacement and bond-line stability. Consequently, the dominant limitation differs between process groups: fusion-based technologies are mainly restricted by thermal degradation phenomena, whereas friction-based techniques are more strongly affected by process reproducibility and local deformation control.

Adhesive selection remains another important challenge. Epoxy, polyurethane, acrylic and thermoplastic systems provide different combinations of stiffness, flexibility and thermal stability; however, none simultaneously satisfies all weld-bonding requirements. High-stiffness epoxy systems generally improve load transfer and fatigue performance but may promote brittle behavior and reduced impact resistance. Polyurethane and acrylic systems provide improved deformability and energy dissipation but usually exhibit lower thermal resistance. Thermoplastic systems offer good processability and vibration damping; nevertheless, their thermal stability and load-bearing capability remain limited under high-temperature welding conditions [18,34,35,36,41].

Future material development should therefore focus on thermally stable and damage-tolerant adhesive systems, including modified epoxy formulations, hybrid polymers, bio-based resins, self-healing materials and advanced thermoplastic systems with improved thermal resistance [33,41,43,51,97,108,109,110,111,112].

Quality control remains another major barrier to wider industrial implementation. The hybrid nature of weld-bonded joints makes defect detection more difficult than in conventional welded or adhesively bonded structures. Typical defects include porosity, local debonding, adhesive degradation, incomplete interaction between the weld and adhesive layer, local adhesive starvation and thermally induced void formation. Conventional ultrasonic and radiographic methods may not always provide sufficient sensitivity, particularly for small interfacial defects. Promising future directions include advanced ultrasonic techniques, active thermography, multi-sensor data fusion, machine-learning-assisted inspection and integrated structural health monitoring systems [101,102,103,104,106,107].

Another limitation is the absence of unified design and qualification methodologies dedicated specifically to weld-bonded joints. Although welding and adhesive bonding are covered by separate standards, their integration still lacks harmonized procedures for fatigue assessment, durability prediction, failure analysis and process validation. This issue becomes particularly important in railway, aerospace and energy-related applications where certification and inspection requirements remain highly restrictive [98,99,100].

Future research should additionally focus on stronger integration between welding technology, adhesive selection, substrate material and mechanical response. The reviewed literature indicates that joint performance depends not only on the welding process itself but also on adhesive family, bond-line thickness, substrate combination and thermal history. Therefore, future studies should increasingly adopt process–adhesive–substrate frameworks rather than investigating these parameters independently.

Further development of predictive methodologies requires coupled thermomechanical–damage models, cohesive zone approaches and experimentally validated simulations capable of describing adhesive degradation, crack initiation, residual stress development and fatigue behavior under realistic service conditions [39,72,81,82,84,85,90,91,113]. In addition to conventional finite element approaches, recent studies have increasingly employed interpretable surrogate modeling, multi-scale mechanical modeling and data-driven fatigue prediction frameworks to accelerate fatigue assessment and reduce computational cost. For example, interpretable random forest surrogate models have been proposed for rapid stress intensity factor (SIF) prediction and fatigue life assessment of welded structures, demonstrating the growing importance of hybrid physics–data methodologies in structural integrity evaluation [114]. Similarly, machine learning and multi-source authentic data-driven frameworks have been applied for fatigue life prediction and structural monitoring of welded systems, indicating increasing interest in predictive maintenance, structural health monitoring (SHM) and digitalized durability assessment [115]. Recent developments in multi-scale mechanical modeling further indicate the increasing role of hierarchical fatigue analysis and damage evolution prediction for improving long-term durability assessment of engineering structures [116]. In addition, artificial intelligence approaches based on Bayesian optimization and CNN–LSTM neural networks have been proposed for fatigue life prediction of welded structures, highlighting the growing importance of AI-assisted durability assessment and data-driven structural integrity analysis [117].

In parallel, digital twins and real-time monitoring are increasingly considered promising tools for process optimization, defect detection and lifetime prediction of weld-bonded structures [104,105,118]. However, implementation of digital-twin frameworks requires process-specific datasets describing coupled thermal, mechanical and damage phenomena occurring during welding and subsequent loading.

For resistance spot weld-bonding (RSWB), relevant monitoring variables include electrode displacement curves, welding current history, dynamic electrical resistance, electrode force and local temperature evolution. These parameters may support prediction of weld nugget formation, adhesive squeeze-out, porosity development and residual stress generation.

For friction stir weld-bonding (FSWB) and friction stir spot weld-bonding (FSSWB), important sensor inputs include tool temperature, rotational speed, axial force, plunge depth and torque response. These variables are particularly useful for modeling material flow, local heat generation, adhesive displacement and damage evolution within the plasticized region.

For laser weld-bonding (LWB), digital-twin models may additionally incorporate laser power stability, melt-pool behavior, thermal field evolution and local temperature distribution. Such datasets may support prediction of adhesive degradation, gas evolution and localized porosity formation near the weld region.

Integration of experimentally acquired sensor data with thermomechanical–damage models and cohesive zone approaches may therefore improve process control, predictive maintenance and long-term durability assessment of weld-bonded structures under industrial operating conditions.

## 8. Conclusions

Weld-bonding represents an important hybrid joining technology for lightweight multi-material structures because it combines localized metallurgical anchoring with the stress redistribution capability, vibration damping and load-sharing effects provided by the adhesive layer. The reviewed literature indicates that weld-bonded joints may provide improved static strength, fatigue performance and energy absorption compared with conventional welded joints, although the extent of improvement strongly depends on process conditions, adhesive type, substrate combination and joint geometry [14,16,22,23,25,27,31,32].

A major conclusion of this review is that the mechanical response of weld-bonded joints cannot be attributed solely to the welding process itself. Joint performance is governed by the interaction between welding technology, adhesive family, bond-line thickness, thermal history and substrate material. Consequently, future investigations should increasingly adopt process–adhesive–substrate approaches rather than analyzing these parameters independently.

Fusion-based processes, particularly resistance spot weld-bonding (RSWB) and laser weld-bonding (LWB), generally provide high local stiffness and strong load transfer due to metallurgical anchoring. However, their performance may be limited by adhesive degradation, gas evolution, porosity formation, local adhesive starvation and residual stress development generated by concentrated heat input [35,36,37,38,39,40,53,54]. In contrast, friction-based techniques such as friction stir weld-bonding (FSWB) and friction stir spot weld-bonding (FSSWB) provide lower thermal exposure, improved preservation of adhesive integrity and reduced residual stresses, although their performance remains strongly dependent on tool movement, material flow, adhesive displacement and process stability [12,24,26,57,58,59,60,61,62,66].

The reviewed studies further indicate that no single weld-bonding process is universally optimal. RSWB remains the most mature solution for high-volume automotive production and body-in-white structures, whereas FSWB and FSSWB are particularly attractive for lightweight aluminum assemblies, battery systems and thermally sensitive structures. LWB offers high dimensional precision and localized heat input but requires strict control of adhesive thermal stability and defect formation [11,26,53,64,74,75,76,77,78,79,80].

Increasing industrial interest in weld-bonding is additionally associated with lightweight multi-material systems, including aluminum–steel assemblies, battery enclosures and composite–metal transition joints used in aerospace structures. In such applications, the adhesive layer contributes not only to load transfer but also to compensation of stiffness mismatch, vibration damping and partial mitigation of thermal expansion differences.

Future development of weld-bonding technologies requires progress in several interconnected areas, including thermally stable and damage-tolerant adhesive systems, low-heat-input joining processes, reliable non-destructive evaluation methods, advanced thermomechanical–damage models and experimentally validated durability prediction approaches. Particular attention should be paid to adhesive degradation, fatigue crack initiation, environmentally assisted degradation, internal defect detection and long-term service performance Recent developments in surrogate modeling, machine learning and digital-twin-assisted fatigue prediction additionally indicate promising directions for future durability assessment and process optimization of weld-bonded structures [18,35,36,42,72,102,103,104,114,115,116,117,118].

The implementation of digital twins, real-time monitoring and structural health monitoring systems may further support predictive maintenance, process optimization and lifetime assessment of weld-bonded structures. Future digital frameworks should increasingly integrate sensor-derived process variables with thermomechanical and cohesive-zone models in order to improve prediction of defect evolution and service behavior.

Overall, weld-bonding should not be considered merely as a combination of welding and adhesive bonding, but rather as a distinct hybrid joining technology with its own process limitations, damage mechanisms, design rules and application potential. Its broader industrial implementation will depend on the integration of material development, process optimization, modeling tools, quality control strategies and standardization procedures.

## Figures and Tables

**Table 1:** Comparative characteristics of adhesive systems used in weld-bonding applications, including thermal stability, mechanical behavior and interaction with welding conditions.

Adhesive Type	Typical Thermal Stability/Service Temperature	Mechanical Characteristics	Behavior Under Welding Conditions	Most Suitable Weld-Bonding Processes	Main Advantages	Main Limitations
**Epoxy**	Stable up to ~180–200 °C [37,38,39,40]	High stiffness, high static and fatigue strength [41,42]	Good thermal stability; possible local carbonization, adhesive degradation, brittleness and gas evolution under excessive heat input [35,36,37,38,39,40]	RSWB, LWB, FSWB	High load-bearing capacity, good durability	Limited deformability, brittle failure tendency, possible delamination
**Polyurethane**	Typically stable below ~120–150 °C depending on formulation [34,43,44,45,46,47]	High flexibility, high energy absorption and impact resistance [15,17,34]	Susceptible to thermal degradation, softening and outgassing at elevated temperatures [34,40,44,45,46,47]	FSSWB, low-heat-input RSWB, FSWB	Good vibration damping and damage tolerance	Lower stiffness and reduced thermal stability compared with structural epoxy systems
**Acrylic**	Moderate thermal stability (~120–180 °C) [35,48,49,50]	Balanced stiffness and flexibility; good dynamic performance [48,50]	Fast curing; moderate resistance to thermal exposure; possible softening under high heat input [35,48,49]	FSWB, FSSWB	Fast curing, good adhesion to metals	Lower thermal stability compared with epoxy adhesives
**Thermoplastic**	Usually below ~100–140 °C depending on polymer type and formulation [17,51,52]	High deformability, good vibration damping capability and relatively low structural stiffness	Local softening, melting, creep deformation and bond-line instability may occur [37,38,39,40,51,52]	Auxiliary and low-load applications	Ease of processing, low cost	Susceptibility to thermal softening and creep; limited applicability in high-temperature weld-bonding processes

Abbreviations: RSWB—resistance spot weld-bonding; FSWB—friction stir weld-bonding; FSSWB—friction stir spot weld-bonding; LWB—laser weld-bonding.

**Table 2:** Comparative characteristics of major weld-bonding processes in terms of thermal conditions, adhesive preservation, defect formation and structural performance.

Process	Process Type	Thermal Conditions	Adhesive Preservation	Typical Thermal/Structural Effects	Typical Defects	Structural Performance
**RSWB**	Fusion-based	Local fusion; temperatures exceeding melting point locally [37,38,39,64]	Moderate to low adhesive preservation; local squeeze-out and thermal degradation may occur [37,38,39,40,54,56,64,65,66,67,68,69]	Distinct HAZ and localized residual stresses [14,31,32,64,65,66,67,68]	Outgassing, porosity, adhesive degradation [37,38,39,40,53,64,65,66,67,68]	High static strength and good fatigue performance when process parameters are optimized [27,31,64]
**FSWB**	Solid-state	~0.6–0.8 melting temperature of the base material [11,12,59,60,61]	High adhesive preservation due to reduced thermal exposure [12,24,26,57,58,59,60,61,62]	Limited HAZ and low thermal distortion [11,12,24,57,58,59,60,61,62]	Minor adhesive degradation, local adhesive flow instability [12,24,57,58,59,60,61,62]	High fatigue resistance and low residual stresses [24,26,57,58,59,60,61,62]
**FSSWB**	Solid-state	Localized frictional heating [12,24,57,58,59,60,61,62,70,71]	High adhesive preservation; suitable for lightweight structures because of limited thermal exposure [12,24,25,26,27,57,58,59,60,61,62]	Localized plastically affected zone [66,70,71,72,73]	Local adhesive displacement and incomplete interfacial bonding [24,26,64]	Often lower load-bearing capacity compared with continuous weld-bonded joints [24,26,64,70,71,72,73]
**LWB**	Fusion-based	Highly concentrated local heating [37,38,39,40,55,74]	Moderate to low adhesive preservation due to concentrated heat input and local adhesive degradation [37,38,39,40,53,55]	Narrow HAZ with steep thermal gradients [53,74,75,76,77,78,79,80,81,82]	Porosity, gas evolution, adhesive decomposition [37,38,39,40,53,55]	High dimensional precision under controlled processing conditions [55,74,75,76,77,78,79,80]

**Table 3:** Comparative mechanical response and dominant failure characteristics of selected weld-bonding processes.

Process	Static Load Transfer Characteristics	Fatigue Response	Dominant Failure Mode	Mechanical Limitations	Typical Mechanical Advantages
**RSWB**	High local stiffness; tensile–shear strength increased by ~40% (DP590) and ~58% (DP780) relative to spot-welded joints and by ~15% and ~39% relative to adhesive-bonded joints [83]	Sensitive to residual stresses and nugget defects; improved endurance limit at 10^6^ cycles compared with spot-welded joints reported for DP steels [83]	Crack initiation frequently observed near the weld nugget; mixed adhesive–metal failure and interfacial fracture reported under cyclic loading conditions [67,69,82]	Stress concentration near the weld nugget and weld–adhesive interface caused by stiffness mismatch [67,69,82,84,85]	High local load-bearing capacity associated with metallurgical anchoring and adhesive-assisted load transfer [31,68,83]
**FSWB**	More uniform stress distribution; average joint efficiency ~73.8%, maximum ~85.2% relative to AA6082-T6 base material [86]	Improved fatigue performance; fatigue strength at 10^6^ cycles reached ~79.9% of adhesive-bonded joints compared with ~41.6% for overlap FSW joints [26]	Distributed damage within adhesive layer and stir zone; crack propagation shifted away from localized weld-edge regions [26,64]	Sensitive to tool rotation, welding speed and adhesive displacement; local hook defects and bond-line instability may reduce fatigue performance [27,57,58,64]	Low residual stresses; fatigue strength at 10^6^ cycles increased from ~41.6% (FSW overlap) to ~79.9% (FSWB) relative to adhesive joints [26]
**FSSWB**	Localized load transfer; tensile–shear failure load increased by 2.7× and stiffness by 1.1× compared with conventional FSSW joints for flow-in FSSWB configurations [66]	Improved resistance to interfacial cracking; failure energy increased by 8.14× relative to FSSW joints [66]	Local cracking within the plastically affected region; crack propagation shifted from weld edge toward adhesive layer in hybrid joints [66]	Lower load-bearing capacity than continuous FSWB systems due to localized geometry and reduced effective bonded area [24,26,64]	Reduced interfacial stress concentration; peak stress decreased from 243.1 MPa to 15.5 MPa under 2 kN loading [66]
**LWB**	High local precision and concentrated load transfer; tensile–shear loads up to ~5.6 kN and strength improvements of ~25% have been reported for representative laser spot joining systems [87]	Fatigue response strongly dependent on adhesive integrity and process optimization; adhesive-bonded joints exhibited up to ~5-fold higher fatigue strength than laser-welded joints, whereas weld-bonded configurations showed intermediate fatigue behavior [88,89]	Interfacial defects, localized fracture and porosity associated with adhesive decomposition and gas evolution near the weld region [40,53,55]	Sensitive to steep thermal gradients, adhesive degradation, gas-induced porosity and local weld instability [40,53,55]	High dimensional accuracy, narrow HAZ and localized heat input resulting in reduced global distortion [55,74,75,76,77,78,79]

## Data Availability

No new data were created or analyzed in this study. Data sharing is not applicable to this article.
